# Exposure to Bacterial and Fungal Aerosols: Microorganism Indices in A Waste-Sorting Plant in Poland

**DOI:** 10.3390/ijerph16183308

**Published:** 2019-09-09

**Authors:** Ewa Brągoszewska

**Affiliations:** Department of Technologies and Installations for Waste Management, Faculty of Power and Environmental Engineering, Silesian University of Technology, 18 Konarskiego St., 44-100 Gliwice, Poland; Ewa.Bragoszewska@polsl.pl; Tel.: +48-322-372-762

**Keywords:** bacterial aerosol, fungal aerosol, sorting plant, indoor air quality, microbiological indices

## Abstract

An increased understanding of airborne microorganism populations should enable a better interpretation of bioaerosol exposure found in a working environment. An assessment of the contamination levels of mesophilic bacterial aerosol (MBA) and fungal aerosol (FA) was carried out using two evaluation indices for microbiological pollution—the total index of microbiological contamination per cubic meter (TIMC/m^3^) and the indoor–outdoor index (IOI). An advantage of selected indices is the inclusion of several co-existing factors that have an impact on the formation of bioaerosol. When properly used, they also highlight the low efficiency of the ventilation system caused by an insufficient air exchange. In this study, the microbial air quality (MAQ) of the working environment was assessed during the spring season at a sorting plant located in Southern Poland. Sampling was undertaken in the plant using an Andersen six-stage impactor which allows the obtainment of information about the size distribution of the air microflora. The value of average concentrations of MBA and the average concentration of FA collected in the preliminary cabin of the sorting plant (PCSP) and the cleaning cabin of the sorting plant (CCSP) were analyzed. The obtained values of MBA were 1.6 times higher indoors, compared to outdoors, while FA was 1.7 times higher outdoors than indoors. The maximum TIMC/m^3^ value was obtained in PCSP (2626). The calculated IOI in this study suggests that MBA concentrations are influenced by internal sources, as opposed to FA. The purpose of this work was to present the usefulness of using indices in assessing air quality.

## 1. Introduction

Indoor air pollution is an important problem because people inhale 6–10 litres of air per minute, which amounts to 15,000 litres of air per day [1] and the health risks from exposure to poor indoor air quality (IAQ) may be greater than those related to outdoor pollution because we spend ~90% of the day in indoor environments, of which ~25% is spent at work [2,3,4,5,6]. Therefore, the evaluation of microbiological air quality (MAQ) and the monitoring of the efficiency of the ventilation system are important aspects in the working environment. The ventilation system has an important role in maintaining proper IAQ in the work environment by transferring the indoor pollutants from a building [7]. Generally, the indoor air environment is closely related to ventilation systems, which are deemed as the ‘respiratory systems’ of buildings, regulating indoor parameters (temperature, humidity, velocity) and cleanliness [8].

Exposure to MAQ in the work environment is associated with a wide range of health effects, including three major groups of diseases—infections and toxic and allergic reactions [9,10,11,12].

The main source of bacterial aerosols in enclosed spaces can be deemed to be human and animal organisms [13,14,15,16,17]. Significant amounts of bacteria are also found in settled dust, where they can be resuspended in the air as a result of secondary particulate emissions, for example, by moving the users’ premises. Even for fungi (of which humans do not appear to be a major primary source), human activities play an important role, for example, in shedding particulate matter from clothing or in suspending settled dust that can contain materials of a fungal origin [18]. However, fungi usually enter a building through outdoor air being extracted through heating, ventilation, and air conditioning systems; via doors and windows; and as contaminants in building materials [19,20,21]. 

In Poland, legislation governing MAQ standards has not been developed and implemented. The main reason for this is a huge variety of air microflora and a large variety of collection methods [16,22]. The harmfulness of biological factors in the Polish regulatory context is set out in the regulation dated 22 April 2005 on harmful biological factors for health in the work environment and the health protection of employees exposed to these factors [23]. On the other hand, the employer is obliged to assess occupational risk [24]. Workers in waste-sorting plants represent a group exposed to a particularly high health-risk associated with the presence of high concentrations of fungi and mesophilic bacteria [25]. Moreover, the workers who have no knowledge of the health hazards are more vulnerable, and coupled with their lack of safety clothing, there could be a risk to their safety and health [26]. There is increasing evidence that shows associations between working in sorting plants and health problems such as allergies, irritation, inflammation, and pulmonary diseases [27].

The subjects of this study were the following—(a) the concentration levels and the size distributions of mesophilic bacterial aerosol (MBA) and fungal aerosol (FA) during the spring season, in a sorting plant located in Southern Poland, (b) which were evaluated by applying the following indices—the total index of microbiological contamination per cubic meter (TIMC/m^3^) and the indoor–outdoor index (IOI). The spring season was selected for this study because recent research of MAQ conducted in Southern Poland indicated that the highest microbial concentration was consistently found in spring [9,28,29]. The TIMC, including the presence of different types of microorganisms, could be a simple method for evaluating the potential biological risk in indoor and outdoor environments. It also allows monitoring the sources of microbial contamination [30,31]. The IOI shows us where the source of a bioaerosol might be found [32]. 

## 2. Materials and methods 

### 2.1. Sampling Site 

**Figure 1:**
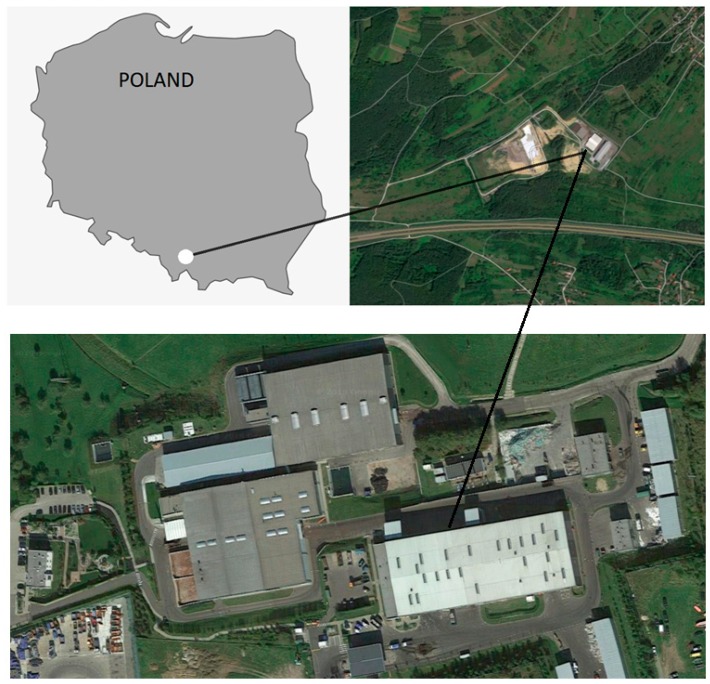
Sampling sites (map source: Google Earth).

The research was carried out at a preliminary cabin of a sorting plant (PCSP) and a cleaning cabin of a sorting plant (CCSP) for mixed municipal waste, located in Southern Poland. The research was conducted during March 2019, in the sorting plant as well as outside the building (Figure 1). Every measurement was conducted between 12:00 and 15:00 from the outdoor air, and from the PCSP and the CCSP, when the average outdoor air temperature was about 12 °C and the indoor temperature was about 17 °C, as well as when the outdoor relative humidity (RH) was about 25%–28%, while the indoor RH was about 15%–20%. The samples of bioaerosols were collected at a height of about 1.5 m to simulate aspiration from the human breathing zone. 

The sorting plant, which had a capacity of 70,000 megagram/year, worked on a two-shift system and was equipped with technology that was adapted for segregating selectively collected municipal waste. The volume of the PCSP was ~178 m^3^ and the volume of the CCSP was ~565 m^3^. Both the PCSP and CCSP had a 20-fold air exchange per hour. Supply and exhaust ventilation was provided, and the air in the cabins was drawn from the conveyor belts. In each sorting cabin (PCSP and CCSP), there were between 6 and 10 people working.

### 2.2. Sampling and Analysis Methods 

**Figure 2:**
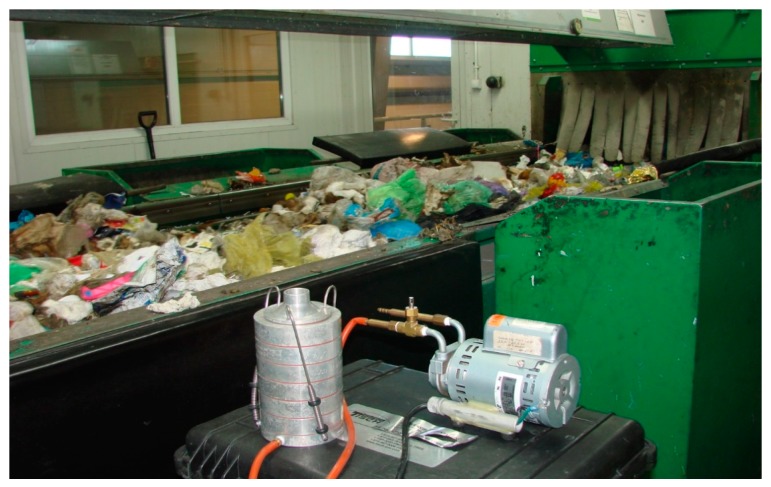
Six-stage Andersen impactor used during measurements in a waste-sorting plant located in Southern Poland.

Measurements of the MBA and FA concentrations were conducted using a six-stage Andersen impactor with cut-off diameters of 7.0, 4.7, 3.3, 2.1, 1.1 and 0.65 µm (Figure 2). During the measurements, the air flow was 28.3 dm^3^/min and the sampling time, calculated following Nevalainen et al. [33], was 10 min. Before and after sampling, the flow rate was measured using a rotameter. Tryptic soy agar (TSA) was used for bacteria, with addition of cycloheximide to inhibit fungal growth. Malt extract agar (MEA 2%) was applied for the fungi, with chloramphenicol added to inhibit bacterial growth. The Petri dishes were incubated for 48 h at 36 ± 1 °C for the mesophilic bacteria and five to six days at 26 °C for fungi. 

The medium was prepared and sterilized in an autoclave prior to pouring it into the Petri dishes. Before and between sampling, the impactor was sterilized using methyl alcohol and periodically cleaned using an ultrasonic cleaner.

The enumeration of microorganisms was conducted according to the Polish standard. Total colony counts were corrected for multiple impactions by the positive-hole method and were expressed as colony-forming units (CFUs) per cubic meter of air [34].

Quality control was practised using the PN-EN12322 [35] and ISO 11133 [36] standards, with the same operation details as in our previous studies [29].

The assessment of bioaerosol contamination was effected using a special indices—TIMC/m^3^ (which is the sum of the values of the total microbial counts determined for the mesophilic bacteria and the fungi) and the IOI resulting from the ratio between the MBA, FA and TIMC/m^3^ values measured inside the building and those measured outdoor.

### 2.3. Statistical Analysis

All statistical analyses were performed using the statistical package Statistica 12 (StatSoft). The concentration values were presented as mean values and standard deviation. The data were not normally distributed, so the non-parametric method was employed. Due to a non-parametric distribution of the collected data (analysed by the Shapiro–Wilk test), the Mann–Whitney U test was applied to assess the differences in the sorting plant cabins. A statistical significance level of α = 0.05 (*p* < 0.05) was used throughout the study.

## 3. Results and Discussion

### 3.1. Levels of MBA, FA and TIMC/m^3^

Table 1 shows the levels of MBA concentration in the indoor air of the sorting plant and the outdoor areas. The mean value of the average concentration of the MBA was the highest in the PCSP (1816 CFU/m^3^). The Mann-Whitney U test confirmed significant differences between MBA concentrations in the PCSP–CCSP outdoor-air of the sorting plant, with *p* < 0.05. The difference between the concentrations of MBA found outside the buildings and in the indoor air was statistically significant (*p* = 0.006). The indoor concentration of mesophilic bacterial aerosol between the different cabins was also statistically significant (*p* = 0.003).

The results obtained in Bydgoszcz, Poland, showed that the level of MBA in a waste-sorting plant during the spring season was 15,200 CFU/m^3^ [37]. The average exposure to MBA in a Korean sorting plant was 190,000 CFU/m^3^ [38]. Studies carried out during different seasons in a sorting plant in Finland, showed that the MBA in the indoor air of the sorting plant ranged from 480 to 1430 CFU/m^3^ [39]. 

Table 2 shows the results of the fungi concentration isolated from the indoor and the outdoor air of the sorting plant. The mean value of the average concentration of FA was the highest in the outdoor air (1221 CFU/m^3^). The Mann–Whitney U test confirmed significant differences between the FA concentration in the indoor and the outdoor air, with *p* < 0.05. The difference between the concentrations of FA found outside the buildings and in the indoor air was statistically significant (*p* = 0.002). The indoor concentration of FA between the different cabins was not significantly different (*p* = 0.61).

The availability of water in the material in the indoor air [40] and relative humidity of outdoor air (RH) are generally the most important environmental factors influencing the concentration level of fungal aerosol. This is because FA favour high moisture and moderate temperatures, while a low water activity, RH and extreme temperatures inhibit growth and spore germination [41]. Zuraimi et al. [42] in Singapore observed higher concentration of FA during rainy weather (2930 CFU/m^3^) than when it was dry (1424 CFU/m^3^). The microbial activity of bioaerosols will be inhibited if the moisture status of air (RH) is too low because a dry environment depresses the metabolism and physiological activities of microorganisms [9,28,43]. In addition, a high moisture status of air (RH) might result in the clumping of cells, which possibly increases the odds of cell survival [44].

Table 3 shows the calculation of the TIMC/m^3^, confirming the presence of higher values of contamination in indoor air in the PCSP, compared to the CCSP. The results of the TIMC/m^3^ (bacteria and fungi) collected from the sorting plant show that the maximum value was in the PCSP (2626), moreover the high value was obtained in the outdoor air (2366). In a waste sorting plant in Cracow, Poland, the value of TIMC/m^3^ in outdoor air was even 61,300 while in indoor 30,490 [45]. Studies carried out in April in a sorting plant located in Korea, showed that the TIMC in indoor air was 21,0000 [38].

Previously conducted studies have shown that the size of the TIMC/m^3^ in the outdoor air is different, depending on the prevailing season and climatic conditions [28,46,47,48]. In the Upper Silesia Region of Poland, the TIMC/m^3^ in outdoor air values were ranged from 340 to 923 during winter and from 1271 to 2977 during the spring season [49]. In Korea, the highest TIMC/m^3^ in atmospheric air was observed in the summer season (5400) and the lowest was observed in winter (580) [50]. The TIMC/m^3^ is a useful index in determining the total counts of microorganisms during environmental monitoring because they have different significances and allow for a more complete evaluation of bioaerosol contamination [31].

In Poland, there are no generally valid values for acceptable concentrations of MBA and FA on a universal scale. In 2004, a Team of Experts on Biological Factors of the Interministerial Commission for Maximum Permissible Concentrations and Strengths of Factors Detrimental to Health in the Work Environment proposed the adoption of recommended values for permissible concentrations of the most common categories of microorganisms and bacterial endotoxins for the air in workplaces (MBA of 1 x 10^5^ CFU/m^3^, FA 5 x 10^4^) [51]. It could be seen that the concentration levels of the obtained MBA and FA in our study were below the proposed standard. However, long-term inhalation of airborne bacteria and fungi in this environment could cause some adverse health effects, especially among those sensitized to this type of air pollution

The IOI shows us where the source of bioaerosol might be found. The average IOI calculated for all indoor and outdoor bacteria concentrations was the highest for the MBA in the PCSP (1.6) (Table 1) and the lowest was for the FA in the CCSP (0.59). When the IOI was > 1, it could be clearly concluded that the major sources of bioaerosols were internal sources. 

In the studied environment, the calculated IOI suggested that the MBA concentrations were influenced by internal sources, as opposed to FA. The IOI for FA was mostly < 1, which suggested that the indoor inhalation exposure to FA was largely influenced by outdoor airborne fungal concentrations and it could be concluded that there was no significant mould source in PCSP and CCSP [48,52]. Outdoor air markedly influences the prevalence of fungal spore levels in indoor air and, thus, it is the major source of fungal infections in indoor environments [53]. Indoors where there is adequate thermal insulation, moisture control and healthy room conditions, fungal spores normally have no chance to colonize [54]. However, the FA concentrations can be higher in buildings with moisture problems than in reference buildings [55]. 

### 3.2. The Size Distribution of MBA and FA

**Figure 3:**
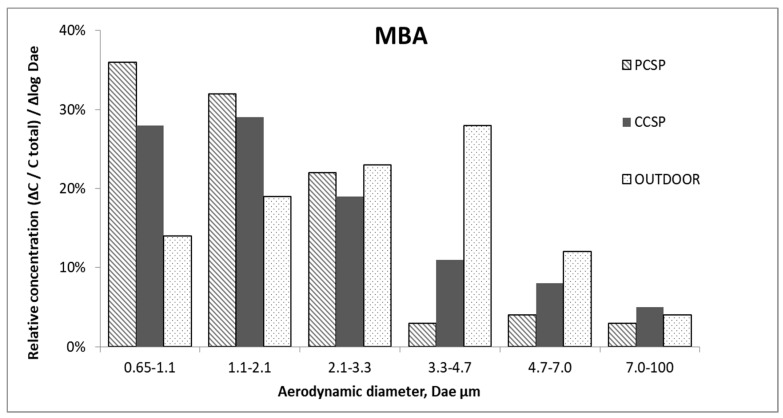
The size distribution of MBA in the preliminary cabin of the sorting plant (PCSP), the cleaning cabin of the sorting plant (CCSP) and outdoor air.

Figure 3 presents the analysis of the number and aerodynamic diameter of MBA collected from the different stages of the impactor in the PCSP and CCSP of a waste-sorting plant, and from outside the building. 

It can be seen that the size distribution of MBA in the indoor air were characterized by a large share of the respirable fraction (the particles less than 3.3 µm). The shape of size distributions might indicate that the particles of culturable bacteria were relatively fresh, and mostly of waste-sorting handled origin. Such results could indicate the existence of a serious potential risk of exposure to particles of respirable sizes (especially for workers), which might reach the trachea, bronchi and alveoli, and contribute to adverse symptoms in the respiratory system [56,57].

**Figure 4:**
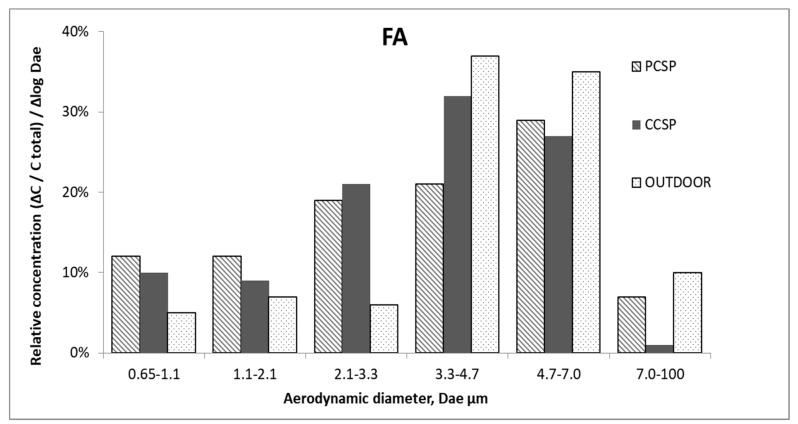
The size distribution of FA in the PCSP, CCSP and outdoor air.

The size distribution for MBA obtained outdoor were unimodal, with a peak falling in the range of particle bacterial aerodynamic diameters, in the range 3.3–4.7 µm. Temporarily, bacterial particles with aerodynamic diameters in the range of 2.1 to 3.3 µm (although they do not significantly affect the particle size distribution) could significantly alter the ratio of the concentrations of the respirable fraction to fraction coarse particles. Their levels could be subject to strong fluctuations depending on the instantaneous meteorological conditions and on the local structure of bioaerosol. Figure 4 presents the analysis of the number and aerodynamic diameter of FA collected from the six stages of the impactor in the PCSP and CCSP of the waste-sorting plant and outside the building.

The size distributions of FA, both indoor and outdoor, were unimodal, with the peak for the particles having an aerodynamic diameter between 3.3 and 7.0 µm, i.e., it shifted towards coarser particles as compared to MBA. It showed that fungi were present in the analyzed air as fungal aggregates and fungal–dust aggregates. The size distribution could also be influenced by the attachment of fungal particles to other particulate matter [58]. The same results were obtained in a sorting plant in Cracow, Poland where the fungal concentration had maximum values in range of 3.3 to 7.0 µm [45].

## 4. Conclusions

The simultaneous study of MBA and FA in indoor and outdoor environments was carried out at a sorting plant in Southern Poland. The average concentration of MBA collected in the PCSP and the CCSP was 1816 CFU/m^3^ and 1254 CFU/m^3^, respectively, and the average concentration of FA collected in the PCSP and CCSP was 810 CFU/m^3^ and 724 CFU/m^3^, respectively. The obtained values of MBA were 1.6 times higher in indoor than outdoor air, while FA was 1.7 times higher outdoors than indoors. The higher outdoor concentrations of FA could reflect better conditions for growing fungi (especially with favourable RH). 

The results of the TIMC/m^3^ (bacteria and fungi) collected from the sorting plant showed that the maximum value was in the PCSP (2626), whereas the high value was obtained also in the outdoor air (2366). The average IOI calculated for all indoor and outdoor bacteria concentrations was the highest for the MBA, in the PCSP (1.6) (Table 1) and the lowest for the FA was in the CCSP (0.59). The indices determined in this study had different advantages—they considered several factors which contributed to the development of different types of microorganisms (TIMC/m^3^), as well as suggested an internal source of bacterial contamination in the building, e.g., handled waste (as per the IOI). The size distribution of MBA indicated that biological particles less than 3.3 µm contributed more than 70% of the total indoor concentration of bacterial aerosol, increasing the health risk for exposed personnel. These results might indicate that the particles of MBA were relatively fresh, and mostly of waste-sorting-handled origin. The FA particles bigger than 3.3 µm contributed more than 50% of the total concentration of FA both indoor and outdoor. These results showed that the FA were present in the analyzed air as fungal aggregates and fungal–dust aggregates. Additionally, the effect of the particle size was also seen in the IOI, where the smaller the size of microorganisms, the higher the IOI [48].

In waste-sorting plants, microbial decomposition of organic material occurred under intensified conditions. This was the reason why bioaerosol emissions from waste treatment facilities are an issue of both occupational health and safety, as well as environmental hygiene aspects [59]. Research has shown particularly high concentrations of bioaerosols that are harmful to employees in the preliminary manual sorting cabin (PCSP) during mixed municipal waste-sorting. Therefore, source control is the first line of defence to protect workers. It is also important to use personal protective equipment (mask with a biological filter FP2, footwear, protective clothing and gloves) and effective and efficient ventilation [25]. Exposure in waste-sorting plants is unavoidable in that such environments constitute a significant threat to the health of workers. Total elimination of many anthropogenic sources is not possible, but important findings of this study could be used to develop realistic management policies and methods, to improve the MAQ.

## Figures and Tables

**Table 1:** Average concentration of mesophilic bacterial aerosol (MBA) colony-forming units (CFUs) per cubic meter (CFU/m^3^) and the indoor–outdoor index (IOI) of the air inside and outside the sorting plant.

MBA					
Location	Average Concentration	SD	Min	Max	IOI
PCSP	1816	810	1488	2711	1.6
CCSP	1254	624	856	1921	1.1
Outdoor air	1138	510	692	1714	-

SD: Standard deviation

**Table 2:** Average concentration of fungal aerosol (FA) CFUs per cubic meter (CFU/m^3^) and the IOI of the air inside and outside the sorting plant.

FA					
Location	Average Concentration	SD	Min	Max	IOI
PCSP	810	401	314	1237	0.66
CCSP	724	316	211	1002	0.59
Outdoor air	1221	911	806	2512	-

SD: Standard deviation

**Table 3:** TIMC/m^3^ and the IOI.

Location	TIMC/m^3^	SD	IOI
PCSP	2626	1100	1.1
CCSP	1978	836	0.84
Outdoor air	2366	1384	-

SD: Standard deviation
